# Unusual Stroke-Like Symptoms in a Patient With Generalized Osteoarthritis

**DOI:** 10.7759/cureus.15951

**Published:** 2021-06-27

**Authors:** Andrew R Medvec, Sanjeev Shrestha, Lisa L Schroeder

**Affiliations:** 1 Internal Medicine, Geisinger Medical Center, Danville, USA; 2 Rheumatology, Geisinger Medical Center, Danville, USA

**Keywords:** isolated nerve palsy, osteoarthritis, isolated hypoglossal nerve palsy, stroke-like symptoms, articular cyst, articular osteophyte, nerve injury

## Abstract

A usual presenting symptom for osteoarthritis (OA) is pain. However, OA of the spine can present as isolated nerve palsy. We present a case of isolated hypoglossal nerve palsy secondary to chronic OA of the cervical spine. A 68-year-old female presented to the emergency department with stroke-like symptoms of three-day duration. History revealed heaviness of the tongue with dysphagia to solid foods, tongue deviation to the right, and slurred speech over the past year. On examination, she had severe OA of the distal and proximal interphalangeal joints. Various imaging modalities revealed isolated right unilateral hypoglossal nerve paralysis secondary to craniocervical junction degenerative disease from C1-occipital osteophyte and juxta-articular atlantooccipital (AO) synovial cyst. This case is unique as evidenced by various imaging modalities which consistently revealed advanced OA of our patient’s AO joint leading to osteophytic and juxta-articular cyst development causing unilateral hypoglossal nerve palsy.

## Introduction

Osteoarthritis (OA) is extremely common in the United States affecting over 32 million people [1]. OA has been considered a “wear and tear” joint disease affecting mostly the elderly population involving the axial (cervical and lumbar spine) and peripheral joints (knees, hips, proximal, and distal interphalangeal joints). Prevalence of cervical facet OA is around 19% in adults aged 45-64 years and 57% in adults aged 65 and older [2]. Proinflammatory factors and proteolytic enzymes causing destruction of the extracellular matrix are the pathogenesis of joint destruction [3]. OA can form osteophytes at the insertion site of tendons and ligaments through the thickening of subchondral bone due to collagen deposits [4]. Osteophytes can cause radicular symptoms including pain, numbness, and weakness with compressions. Although pain is the initial most common presentation, OA of the spine can present with isolated nerve injury. Here, we present a case of isolated hypoglossal nerve palsy secondary to chronic OA of the cervical spine.

## Case presentation

A 68-year-old female presented to the emergency department (ED) with a three-day history of worsening right-sided throbbing neck pain, dizziness, and dysarthria. History revealed heaviness of the tongue with dysphagia to solid foods, tongue deviation to the right, and slurred speech over the past year. She was previously evaluated by her primary care physician who ordered a stroke workup with magnetic resonance imaging (MRI) and magnetic resonance angiography (MRA) of her neck. These showed no evidence of an ischemic or hemorrhagic event but revealed significant stenosis in her left external carotid artery with atherosclerotic and plaque buildup. She was started on lisinopril, atorvastatin, and low-dose aspirin. No further interventions were conducted. The patient’s past medical history also revealed extensive generalized OA with bilateral total knee arthroplasties (TKAs) eleven and eight years prior to presentation, and OA of the right acromioclavicular joint currently being treated with cortisone injections and physical therapy.

**Figure 1:**
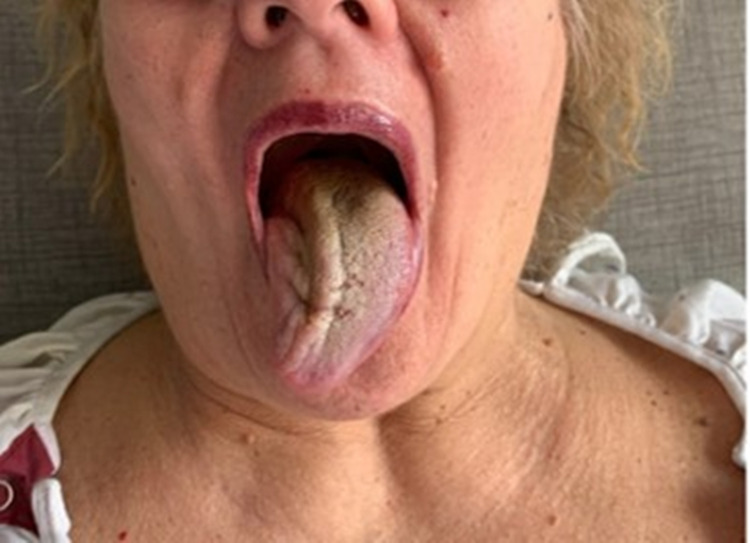
Right-sided deviation and hypotonia of the tongue.

In the ED, she denied otalgia, tinnitus or diminished hearing, hoarseness, and both facial and shoulder weakness. Right deviation and hypotonia of the tongue were evident on examination (Figure 1).

**Figure 2:**
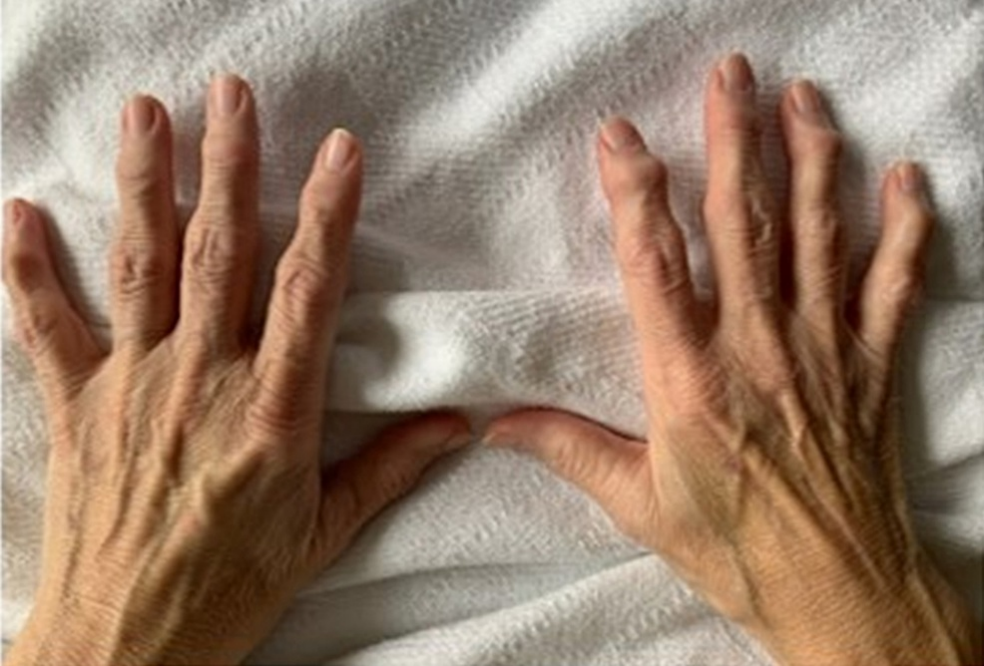
Numerous Heberden’s (distal interphalangeal joint) and Bouchard’s nodes (proximal interphalangeal joint) present on physical examination.

There were no vertiginous, positional, or orthostatic symptoms. Examination of her hands was remarkable for numerous Heberden’s and Bouchard’s nodes (Figure 2).

**Figure 3:**
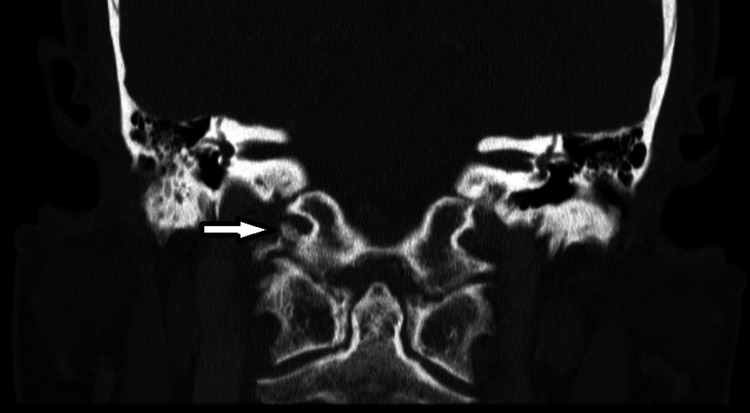
CT coronal section of the head and neck revealing AO osteophyte extending into the right jugular foramen with extrinsic compression and mild stenosis of the right internal carotid artery and jugular bulb. CT: computed tomography; AO: atlantooccipital

A computed tomography angiogram (CTA) head and neck was performed which showed no evidence of acute intracranial hemorrhage, transcortical infarction, or large vessel occlusion. However, CT imaging revealed evidence of an amorphous calcification surrounding the right atlantooccipital (AO) joint inferior to the skull base with extension to the hypoglossal canal and an adjacent low-attenuation/cystic lesion extending into the right jugular foramen with extrinsic compression and mild stenosis of the right internal carotid artery and jugular bulb (Figure 3). Additionally, there was radiographic evidence of chronic denervation atrophy of the right hemi-tongue and styloglossus muscle.

**Figure 4:**
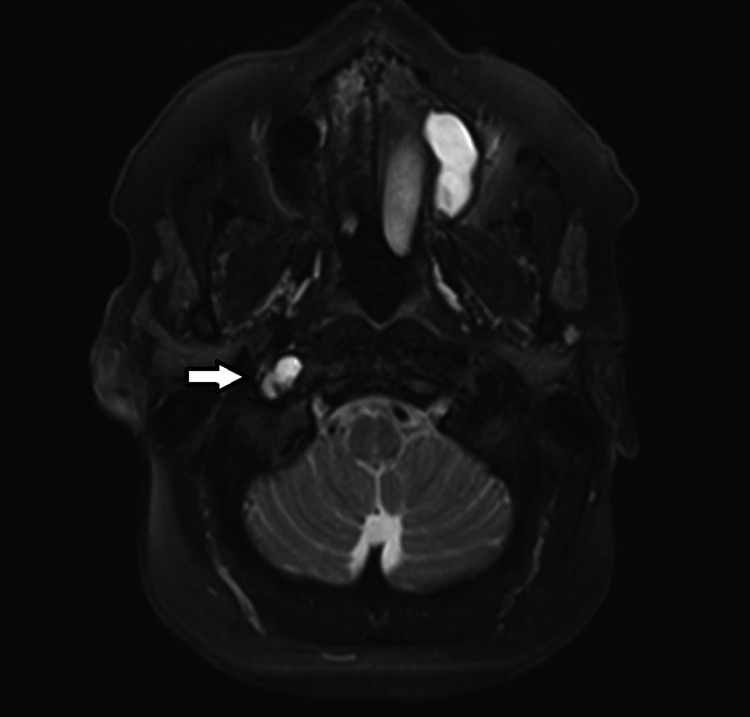
MRI of the soft tissue neck revealing a 1.5-cm cystic lesion inferior to the skull base in the post-styloid parapharyngeal space adjacent to the right AO joint. MRI: magnetic resonance imaging; AO: atlantooccipital

Otolaryngology and neuroradiology were consulted. An MRI of the soft tissue neck and the internal auditory canal (IAC) was recommended and revealed a 1.5-cm cystic lesion (with minimal thin peripheral enhancement) just inferior to the skull base in the post-styloid parapharyngeal space adjacent to the right AO joint with chronic hypoglossal end-organ denervation atrophy (Figure 4). We also identified a left maxillary mucous retention cyst.

**Figure 5:**
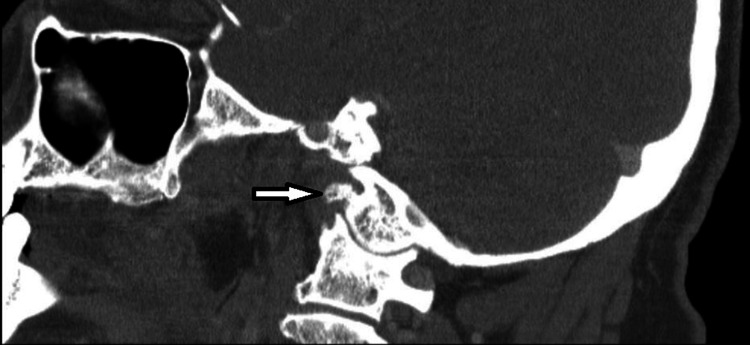
CT of the temporal bone sagittal section showing right AO joint osteophyte with extension into the hypoglossal canal. CT: computed tomography; AO: atlantooccipital

Similar evidence of extrinsic compression of the jugular bulb and cervical internal carotid artery was also seen. There was no evidence of acute ischemic infarction, mass effect, hydrocephalus, or extra-axial fluid collection. No cerebellopontine angle or IAC lesions were identified. A CT of the temporal bone with and without contrast further revealed asymmetric degenerative changes/OA at the right AO joint with amorphous calcification extending to the hypoglossal canal with an adjacent synovial cyst extending into the right jugular foramen (Figure 5).

A bedside flexible laryngoscopy was unremarkable. Fluoroscopic swallow evaluation showed mild oral dysphagia. An audiology evaluation was completed which showed bilateral mild-to-moderate high-frequency sensorineural hearing loss bilaterally.

She was diagnosed with an isolated right unilateral hypoglossal nerve paralysis secondary to craniocervical junction degenerative disease from C1-occipital osteophyte and juxta-articular AO synovial cyst. A dexamethasone taper and gabapentin were started for symptomatic relief. She was subsequently discharged. On outpatient follow-up with otolaryngology, she reported significantly improved right-sided neck pain since initiation of steroids. After finishing her steroid taper, she was continued on gabapentin.

Speech reevaluated our patient finding no sign of aspiration and recommended a regular textured diet. Treatment options were discussed with the patient, including surgical options; however, she decided to ultimately forego surgery and continue conservative management. She continues to follow up as an outpatient with periodic MRI IAC.

## Discussion

The hypoglossal nerve is the 12th cranial nerve whose course originates from the left and right hypoglossal nuclei found in the lower medulla. Upon exiting the medulla, the hypoglossal nerve transverses through the hypoglossal canal of the occipital bone, descending through the neck to the angle of the mandible and then passing underneath the tongue and innervating the intrinsic and extrinsic muscles of the tongue [5]. Isolated hypoglossal nerve palsy without the involvement of any other medullary nerves is extremely uncommon [6]. Isolated injury presents as weakness of the tongue muscles on the affected side, leading to the deviation of the tongue towards the affected side along with speech and swallowing dysfunction. A segmental approach previously described by Thomspon et al. revealed five segments upon injury of the nerve: medullary, cisternal, skull base, nasopharyngeal carotid space, and sublingual segments [7]. A case series reported isolated hypoglossal nerve palsy in nine patients, four of whom were found to have an idiopathic cause with excellent recovery, three were due to metastatic disease affecting the base of the skull, one had a Chiari malformation, and one had an arteriovenous fistula of the transverse sinus [6]. Other reported cases of isolated hypoglossal nerve palsy were induced by trauma (either iatrogenic injury or road traffic accidents) or by a vascular incident such as carotid artery dissection [8]. There have been reports of isolated atlantoaxial synovial cysts causing compression of the hypoglossal nerve [9] and cervical osteophyte causing compression [8,10]. However, to our knowledge, there have been no reports of a combined osteophyte and synovial cyst causing isolated hypoglossal nerve palsy.

The initial presenting features were indicative of a stroke or dissection. Upon presentation, these were ruled out by the initial CTA head and neck. Furthermore, our patient was previously evaluated for an ischemic/hemorrhagic event which was unremarkable. The chronicity of symptoms pointed to other differentials, including a meningioma or nerve sheath tumor, metastasis, trauma, Chiari malformation, or an arteriovenous malformation (AVM). Meningioma or nerve sheath tumor was less likely as MRI of the IAC revealed no radiographic evidence of tumor involvement. Given that the patient denied recent trauma and atrophy and fatty replacement of the tongue were observed, the lesion was thought to be longstanding. Trauma was also excluded through a thorough physical examination and imaging. Chiari malformation and AVMs were not evident on contrast imaging. When evaluating a hypoglossal nerve dysfunction, it is important to recognize the strengths and weaknesses of each imaging modality. MRI has superior soft tissue enhancement, whereas CT imaging has superior cortical bone delineation [7]. Hence, in our case, CT imaging is the preferred method for osseous pathology to discover degenerative OA.

## Conclusions

This case is unique as evidenced by various imaging modalities that consistently revealed advanced OA of our patient’s AO joint leading to osteophytic and juxta-articular cyst development causing unilateral hypoglossal nerve palsy. This was in the setting of generalized OA evident in bilateral hands with Heberden’s and Bouchard’s nodes on examination and a history of bilateral TKAs and OA of the right acromioclavicular joint being treated with cortisone injections and physical therapy. This case, though rare, underscores the importance of broadening the differential diagnosis of a patient who presents with unilateral hypoglossal nerve palsy, especially if there are history and extracranial physical examination findings suggestive of advanced OA.
